# GLP-1R Signaling Directly Activates Arcuate Nucleus Kisspeptin Action in Brain Slices but Does not Rescue Luteinizing Hormone Inhibition in Ovariectomized Mice During Negative Energy Balance

**DOI:** 10.1523/ENEURO.0198-16.2016

**Published:** 2017-01-20

**Authors:** Kristy M. Heppner, Arian F. Baquero, Camdin M. Bennett, Sarah R. Lindsley, Melissa A. Kirigiti, Baylin Bennett, Martha A. Bosch, Aaron J. Mercer, Oline K. Rønnekleiv, Cadence True, Kevin L. Grove, M. Susan Smith

**Affiliations:** 1 Division of Diabetes, Obesity and Metabolism, Oregon National Primate Research Center, Oregon Health & Science University, Beaverton, OR 97006; 2 Department of Physiology and Pharmacology, Oregon Health & Science University, Portland, OR, 97239; 3 Novo Nordisk Research Center, Seattle, WA 98109; 4 Division of Neuroscience, Oregon National Primate Research Center, Oregon Health & Science University, Beaverton, OR 97006

**Keywords:** fasting, GLP-1, hypothalamus, kisspeptin, LH, liraglutide

## Abstract

Kisspeptin (Kiss1) neurons in the hypothalamic arcuate nucleus (ARC) are key components of the hypothalamic-pituitary-gonadal axis, as they regulate the basal pulsatile release of gonadotropin releasing hormone (GnRH). ARC Kiss1 action is dependent on energy status, and unmasking metabolic factors responsible for modulating ARC Kiss1 neurons is of great importance. One possible factor is glucagon-like peptide 1 (GLP-1), an anorexigenic neuropeptide produced by brainstem preproglucagon neurons. Because GLP fiber projections and the GLP-1 receptor (GLP-1R) are abundant in the ARC, we hypothesized that GLP-1R signaling could modulate ARC Kiss1 action. Using ovariectomized mice, we found that GLP-producing fibers come in close apposition with ARC Kiss1 neurons; these neurons also contain *Glp1r* mRNA. Electrophysiological recordings revealed that liraglutide (a long-acting GLP-1R agonist) increased action potential firing and caused a direct membrane depolarization of ARC Kiss1 cells in brain slices. We determined that brainstem preproglucagon mRNA is decreased after a 48-h fast in mice, a negative energy state in which ARC Kiss1 expression and downstream GnRH/luteinizing hormone (LH) release are potently suppressed. However, activation of GLP-1R signaling in fasted mice with liraglutide was not sufficient to prevent LH inhibition. Furthermore, chronic central infusions of the GLP-1R antagonist, exendin(9–39), in *ad libitum*–fed mice did not alter ARC *Kiss1* mRNA or plasma LH. As a whole, these data identify a novel interaction of the GLP-1 system with ARC Kiss1 neurons but indicate that CNS GLP-1R signaling alone is not critical for the maintenance of LH during fasting or normal feeding.

## Significance Statement

Reproductive dysfunction is associated with metabolic imbalance, and identifying the underlying molecular mechanisms linking metabolic status with reproductive function is of great importance. Kisspeptin neurons (Kiss1) located in the arcuate nucleus (ARC) of the hypothalamus are essential for fertility and are potently inhibited during negative energy balance; this inhibition occurs in the presence or absence of ovarian steroids. Preproglucagon-expressing neurons located in the brainstem send abundant fiber projections to the ARC, where they release the anorexigenic neuropeptide glucagon-like peptide 1 (GLP-1). The aim of these studies was to determine the interaction of the CNS GLP-1 system with ARC Kiss1 activity to potentially provide a link between systems that control energy balance with those that control reproductive neuroendocrine output.

## Introduction

Adequate nutrient availability is essential to maintain proper reproductive function, and states of chronic negative energy balance lead to the suppression of the hypothalamic-pituitary-gonadal (HPG) axis. Kisspeptin (Kiss1)-expressing neurons are positive regulators of gonadotropin-releasing hormone (GnRH) release, and Kiss1 signaling through its receptor (Kiss1r) is essential for fertility (de Roux et al., 2003; Seminara et al., 2003). Kiss1 neurons located in the arcuate nucleus (ARC) of the hypothalamus act on GnRH nerve terminals in the median eminence and regulate basal pulsatile GnRH/luteinizing hormone (LH) release (Li et al., 2009; Han et al., 2015). Similar to other ARC neurons, ARC Kiss1 neurons respond to metabolic cues denoting changes in energy status and alter their activity (True et al., 2013; Frazao et al., 2014; Nestor et al., 2014). Therefore, ARC Kiss1 neurons are thought to be key integrators of metabolic status with proper output of GnRH/LH release. However, the identity of nutrient-sensing systems that regulate ARC Kiss1 action in response to changes in energy status remains elusive. Previously, our laboratory used rodent models to show that low leptin and insulin levels associated with negative energy balance do not appear to be responsible for the suppression of GnRH/LH release (Xu et al., 2009; True et al., 2011).

We identified glucagon-like peptide 1 (GLP-1) as a potential nutrient-sensing system that integrates metabolic status with reproductive neuroendocrine function in part through the regulation of ARC Kiss1. Posttranslational processing of the preproglucagon gene (*Gcg*) gives rise to GLP-1 which is mainly produced in the gastrointestinal tract and brain (Sandoval and D’Alessio, 2015). GLP-1 mediates its action through the GLP-1 receptor (GLP-1R), a seven-transmembrane G-protein–coupled receptor (Mayo et al., 2003). Peripherally and centrally administered GLP-1R agonists activate the GLP-1R in the CNS, suppressing feeding and body weight (Heppner and Perez-Tilve, 2015). This effect is mediated in part through activation of anorexigenic neurons that express proopiomelanocortin (POMC) and cocaine- and amphetamine-regulated transcript (CART), as well as inhibition of orexigenic neurons that express neuropeptide Y (NPY) and agouti-related peptide (AgRP) located in the ARC (Secher et al., 2014). Preproglucagon-expressing neuronal cell bodies produce GLP-1, GLP-2, and oxyntomodulin (Larsen et al., 1997) and are confined to the brainstem, whereas their fibers project to numerous areas of the brain. Preproglucagon-expressing neurons project heavily to areas of the hypothalamus that regulate energy metabolism (Llewellyn-Smith et al., 2011; Vrang and Grove, 2011), which is consistent with a functional role of GLP-1 in the regulation of feeding and body weight. One hypothalamic nucleus with the highest levels of preproglucagon-expressing fibers, as well as GLP-1R, is the ARC (Merchenthaler et al., 1999; Llewellyn-Smith et al., 2011; Vrang and Grove, 2011; Rønnekleiv et al., 2014; Heppner et al., 2015). GLP-1–producing neurons are activated in response to calorie ingestion (Kreisler et al., 2014). Furthermore, brainstem preproglucagon expression is inhibited during fasting (Huo et al., 2008) and upregulated during high-fat diet feeding (Knauf et al., 2008), suggesting that preproglucagon neurons act as metabolic fuel sensors.

Reports on the role of GLP-1 in regulating reproductive function, however, are limited. Female *Glp1r* ^–/–^ mice have delayed onset of puberty, suggesting a role for GLP-1R signaling in regulating reproductive function (MacLusky et al., 2000). Functional studies indicate that GLP-1 promotes GnRH secretion from isolated hypothalamic tissue and acts centrally to enhance LH levels in male rats (Beak et al., 1998). More recent studies demonstrate that GLP-1 acts centrally to enhance the preovulatory LH surge of intact female rats (Outeirino-Iglesias et al., 2015). Taken together, these data suggest that GLP-1 promotes GnRH/LH release. Based on the neuroanatomical distribution of GLP-1–producing fibers as well as the functional role of stimulating GnRH/LH release, we hypothesized that GLP-1R signaling activates ARC Kiss1 neurons to enhance GnRH/LH release. Thus, decreased brainstem preproglucagon could contribute to the suppression of ARC Kiss1 activity and suppression of downstream GnRH/LH release during negative energy balance. The aims of these studies were (1) to determine whether the CNS GLP-1 system has neuroanatomical and functional interaction with ARC Kiss1 neurons and 2) to assess whether GLP-1R signaling plays a role in the suppression of the reproductive neuroendocrine axis that occurs during fasting, using an ovariectomized (OVX) mouse model.

## Materials and Methods

### Animals

All animals were fed a standard diet (Purina lab chow; catalog # 5001) and maintained on a 12:12-h light-dark cycle at 22°C with free access to food and water unless noted otherwise. For histology experiments and electrophysiological recordings, *Kiss1*-CreGFP mice on a C57BL/6 background were produced by C.F. Elias and colleagues at the University of Michigan (Cravo et al., 2011) and bred at the facilities at Oregon Health & Science University. For single cell RT-PCR experiments, female *Kiss1*-CreGFP mice (C57BL/6J and S129 background) were originally produced by R.A. Steiner and colleagues at the University of Washington (Gottsch et al., 2011) and then bred at the facilities at Oregon Health & Science University. For studies involving fasting or intracerebroventricular (ICV) infusion, adult female C57BL/6J mice (12–14 weeks old) were purchased from the Jackson Laboratory. For studies involving dual *in situ* hybridization, adult female mice were purchased and ovariectomized at Jackson Laboratory. All animal procedures were approved by Oregon Health & Science University and the Novo Nordisk Research Center Institutional Animal Care and Use Committees.

### Ovariectomy and estradiol replacement

Adult female mice were anesthetized using 2.5% isoflurane in oxygen delivered by a nose cone. After receiving a preoperative dose of carprofen (5 mg/kg), ovaries were removed through bilateral lumbar incisions. The vasculature to the ovary and body wall were sutured, and wound clips were used to close the incision. For surgeries involving estradiol (E_2_) replacement, an E_2_-filled capsule was implanted in the interscapular region immediately after OVX surgery. The E_2_ implants were made of Silastic tubing (0.59 inches long, 0.078 inches inner diameter, 0.125 inches outer diameter; Dow Corning) and filled with a low dose of crystalline E_2_ (20 μg/mL, in sesame oil) as previously described (Navarro et al., 2015).

### Immunohistochemistry

Female *Kiss1*-CreGFP mice (Cravo et al., 2011) underwent OVX surgery and a week later were sedated with ketamine (80 mg/kg) and xylazine (10 mg/kg) and perfused with 4% paraformaldehyde in 0.1 m phosphate (PB) buffer, pH 7.4. Brains were removed and postfixed in 4% paraformaldehyde overnight at 4°C, cryoprotected with 25% sucrose in 0.05 m potassium phosphate-buffered saline (KPBS), and stored at –80°C until sectioning. Sections were cut at 25 μm on a freezing microtome in a one-in-six series. For analysis of preproglucagon-expressing fiber contacts onto Kiss1 neurons, the tissue sections were washed in KPBS several times and preincubated in blocking buffer (KPBS, 0.4% Triton X-100, and 2% normal donkey serum) for 30 min before incubating in chicken anti-GFP (1:5000; Aves Labs; cat. # GFP-1020) and mouse anti–GLP-2 (1:2000; Novo Nordisk) in blocking buffer for 24 h at 4°C. The monoclonal GLP-2 antibody was raised against full-length human GLP-2 (GLP-2_1–33_) and has been shown to have complete overlap with GLP-1 immunostaining ((Tang-Christensen et al., 2000; Vrang et al., 2007). Because of this overlap, no distinction is made whether fibers are GLP-1 or GLP-2; they are referred to as GLP fibers. After washes in KPBS, tissue sections were incubated for 1 h in a cocktail of Alexa Fluor 568 donkey anti-mouse antibody (1:1000; Invitrogen; cat. #A10037) and Alexa Fluor 488 goat anti-chicken (1:1000; Invitrogen; cat. # A11039) at room temperature, washed and mounted on gelatin-coated glass slides, and coverslipped with SlowFade Gold antifade reagent (Invitrogen; cat. #S36936). For analysis of GLP contacts onto GnRH neurons, the same protocol as above was implemented using primary antibodies mouse anti–GLP-2 (1:2000; Novo Nordisk) and rabbit anti-GnRH (1:32,000; EL-14; Ellinwood et al., 1985) and secondary antibodies Alexa Fluor 647 donkey anti-mouse (1:1000; Invitrogen; cat. #A31571) and Alexa Fluor donkey anti–rabbit 568 (1:1000; Invitrogen; cat. #A10042).

### Confocal analysis

Immunofluorescence images were taken with a Leica SP5 confocal microscope with Acousto-Optical Beam Splitter. Analyses of GLP-immunoreactive (GLP-ir) fibers making close appositions to ARC Kiss1 fibers and GnRH cell bodies were performed as previously described (True et al., 2013). Photomicrographs were taken at 40× magnification at 1024 × 1024-pixel resolution and speed of 700 Hz. Focal planes were 1 μm apart for analysis, and four ARC sections and five to seven preoptic areas were analyzed per animal. For more abundant ARC Kiss1 cells, all visible cells in confocal photomicrographs of four ARC sections (unilateral) were analyzed for contact analysis. Stacks were analyzed using ImageJ software (NIH).

### Single-cell RT-PCR

*Kiss1*-CreGFP mice (Gottsch et al., 2011) were OVX bilaterally and killed a week later for tissue collection. Single-cell transcriptomes were isolated from *Kiss1*-CreGFP cells as previously described (Bosch et al., 2013; Navarro et al., 2015). Primers were designed to span at least one intron–exon boundary using Clone Manager software (Sci-Ed Software). Stringent PCR conditions were tested to determine the optimal primer concentration, magnesium concentration, and annealing temperature to produce a single clear band. The primer sequences for *Glp1r* and *Kiss1* were *Glp1r* (149-bp product, accession number NM_021332, forward primer 474–494 nt, reverse primer 602–622 nt); *Kiss1* primers were described previously (Zhang et al., 2013). PCR was performed on 3 μl of cDNA in a 30-μl final volume containing 1× Go *Taq* Flexi buffer (Promega), 2 mm MgCl_2_, 0.33 mm deoxynucleoside triphosphate, 0.33 μm forward and reverse primers, 2 U Go *Taq*, and 0.22 μg TaqStart antibody (Clontech) for 50 cycles of amplification with specific annealing temperatures (*Glp1r*, 60°C; *Kiss1*, 57°C). PCR products were visualized with ethidium bromide on a 2% agarose gel and confirmed by sequencing. As a negative control, artificial CSF samples were collected in the vicinity of the dispersed cells and processed in the RT-PCR assays. Water blanks were also included in each RT-PCR assay. In addition, several single cells were processed in the RT-PCR but without reverse transcriptase (RT) to ensure that genomic DNA was not being amplified. Basal hypothalamic tissue RNA was also included as a positive control (with RT) and negative control (without RT).

For determination of neuronal expression of a particular transcript, 127 neurons (16–33 cells/animal) were harvested from five animals. The number of arcuate Kiss1-GFP neurons expressing *Glp1r* was counted for each animal, and the mean number of neurons/animal was determined and used for further analysis of mean, SEM, and percentage expression.

### Dual *in situ* hybridization

For dual *in situ* hybridization (ISH), formalin-fixed paraffin-embedded brain tissue was cut into 5-μm sections using a rotary microtome (RM2255, Leica Biosystems) and mounted onto Superfrost Plus glass (Thermo Fisher Scientific). We sectioned brains from OVX females (*n* = 4) and sampled the ARC at two distinct anatomical locations within the ARC (–1.3 and –1.8 from bregma). Brain tissues were prepared for RNAscope ISH (ACD Bio) following the manufacturer’s recommendations (ACD Bio; #322452-USM), and duplex chromogenic ISH was executed in a HybEZ System following the protocol from ACD Bio (#322500). Our experiments used probes to Mm-*Glp1r* (#418851-C2) and Mm-*KISS1* (#408001), which were labeled with red and green chromogens, respectively, after signal amplification steps. After staining, slides were counterstained in 20 dips of Mayer’s hematoxylin (Sigma-Aldrich), dried in an oven at 60°C for 2 h, and coverslipped with Ecomount mounting medium (BioCare). Finished slides were scanned at 40× on a Zeiss AxioScan.Z1 for *post hoc* analysis. All representative images were matched for zoom level and brightness/contrast.

### Electrophysiology

All recordings were performed in ARC *Kiss1*-CreGFP neurons (Cravo et al., 2011) at 60–90 d of age. OVX and OVX + E_2_ surgeries were performed 8–10 d before recordings using the methods described above. Coronal slices containing the ARC were prepared as previously described (Qiu et al., 2010). Briefly, brain slices (200 μm) containing ARC were maintained with constant flow (1–2 ml/min) of artificial CSF containing the following (in mm): 124 NaCl, 5 KCl, 2.6 NaH_2_PO_4_, 2 MgSO_4,_ 1 CaCl_2_, 10 HEPES, 10 glucose; oxygenated (95% O_2_, 5% CO_2_) osmolarity ∼300 at 32°C–33°C. For current-clamp experiments, microelectrodes had resistances of 3–6 mΩ and were filled with an internal solution containing the following (in mm): 125 K-gluconate, 2 KCl, 10 EGTA, 5 HEPES, 1 ATP, 0.3 GTP, pH 7.25 with KOH; osmolarity ∼295 mosm/l. Data acquisition was performed using a multiclamp 700B amplifier (Molecular Devices). Data were filtered at 3 KHz and sampled at 5–10 KHz using a computer interface Digidata 1322 and pClamp 9.2 software (Molecular Devices). The liquid junction potential of 5 mV was corrected in the analysis. All solutions were made fresh the day of the experiment. Liraglutide was obtained from Novo Nordisk. 6-Cyano-7-nitroquinoxaline-2,3-dione (CNQX) and dl-2-amino-5-phosphonovaleric acid (AP5) were obtained from Tocris, and tetrodotoxin (TTX) from Alomone Labs.

### Fasting and brainstem dissection

Adult C57BL/6J female mice from the Jackson Laboratory (12–14 weeks) underwent OVX surgery as described above. One week after surgery, animals were maintained on an *ad libitum* diet or fasted for 48 h. We chose a 48-h fast because both brainstem preproglucagon (Huo et al., 2008) and LH levels (Huang et al., 2008) have been reported to be inhibited in mice using this paradigm. After the 48-h fast, all animals were anesthetized with isoflurane and decapitated. The brain was placed into a 1-mm coronal brain matrix and a 3-mm section containing the brainstem was collected (–6 to –9 mm posterior to bregma). The tissue was then frozen immediately on liquid nitrogen and stored at –80°C until RNA extraction was performed.

### Fasting and liraglutide treatment

Adult C57BL/6J female mice from the Jackson Laboratory (12–14 weeks old) underwent OVX surgery as described above. One week after surgery, animals were fed *ad libitum* or had their food removed at 0900. Also at this time, *ad libitum*–fed animals received twice-a-day subcutaneous saline injections and fasted animals received twice-a-day subcutaneous injections of saline or the GLP-1R agonist, liraglutide (30 nmol/kg per injection; Novo Nordisk). Peripheral injection of liraglutide has been reported to penetrate into the ARC (Secher et al., 2014). After 48 h, all animals were anesthetized with isoflurane and decapitated to collect trunk blood.

### Intracerebroventricular exendin(9–39) infusion and arcuate dissection

Adult C57BL/6J female mice from Jackson laboratory (12–14 weeks old) underwent OVX surgery as described above. One week later, animals were stereotaxically implanted (David Kopf Instruments) with a cannula (brain infusion kit #3, Alzet) placed in the lateral cerebral ventricle as previously described (Heppner et al., 2012). A polyethylene catheter attached the cannula to an osmotic minipump (1007D, Alzet) that was subcutaneously implanted. The osmotic minipump infused either saline or exendin(9–39) (Ex-9; 7.5 nmol/d; American Peptide, cat. #46-3-10). After 6 days of ICV infusion, animals were anesthetized with isoflurane and decapitated. Trunk blood was collected, and the brain was dissected out from the skull and placed in a 1-mm coronal brain matrix. The first blade was placed at the caudal extent of the hypothalamus, and the second blade was placed 2 mm rostral. The 2-mm thick coronal slice was placed on a chilled Petri dish, and a dissection razor (Harris Uni-Core; cat. #7093508) was used to collect the ventral aspect of the brain containing the ARC. The tissue was then immediately frozen on liquid nitrogen and stored at –80°C until RNA extraction was performed. Cannula placement was confirmed by increased expression of *Agrp* mRNA in ARC tissue of Ex-9–treated mice compared with saline-treated controls.

### RNA extraction and qPCR for brainstem and arcuate tissue

RNA was isolated using Trizol and the RNeasy micro kit with on-column deoxyribonuclease I treatment (Qiagen). Quality and integrity of RNA was determined using nanodrop spectrophotometer ND-1000. Reverse-transcriptase reactions were prepared using 1 µg of RNA and iScript cDNA Synthesis Kit (Bio-Rad). Quantitative real-time PCR was completed using TaqMan probes (Applied Biosystems) for *Gcg* (Mm01269055_m1), *Agrp* (Mm00475829_g1), *Kiss1* (Mm03058560_m1), and housekeeping gene *18s* (Hs03003631_g1) was used as an endogenous control to normalize each sample and gene. PCRs were in a 10-μl volume using 0.5 μl TaqMan probe, 10 ng cDNA template, 5 μl TaqMan Gene Expression Master Mix II with UNG (Applied Biosystems), and 2.5 μl DNase/RNase-free molecular grade water (Qiagen). Real-time PCR was run using an Applied Biosystems 7900HT Fast Real-Time PCR system with initial denaturing at 50°C for 2 min and 95°C for 10 min, followed by 40 cycles at 95°C for 15 s, and annealing at 60°C for 1 min. Results were calculated using the Pfaffl method (Pfaffl, 2001).

### LH measurements

For experiments involving LH measurements, trunk blood was collected into a tube containing a cocktail of heparin (10 µl of 1000 USP/mL) and protease inhibitor (10 µl of aprotinin 10,000 KIU/ml; Fisher Scientific, cat. #BP2503-10). Plasma was sent to the University of Virginia Center for Research in Reproduction Ligand Assay and Analysis Core (Charlottesville, VA) to be measured for LH by radioimmunoassay.

### Statistical analysis

Statistical analysis was performed using GraphPad Prism, version 6.0. Statistical significance was determined by unpaired Student’s *t*-test, one-way ANOVA followed by Tukey’s multiple comparison or Bonferroni’s correction *post hoc* test, or two-way ANOVA followed by Bonferroni’s multiple comparison *post hoc* test. The statistical analysis for each experiment is stated in the figure legend. All results are given as means ± SEM. Results were considered statistically significant when *p* < 0.05.

## Results

### Neuroanatomical interaction of the CNS GLP system with ARC Kiss1 cells

**Figure 1.**
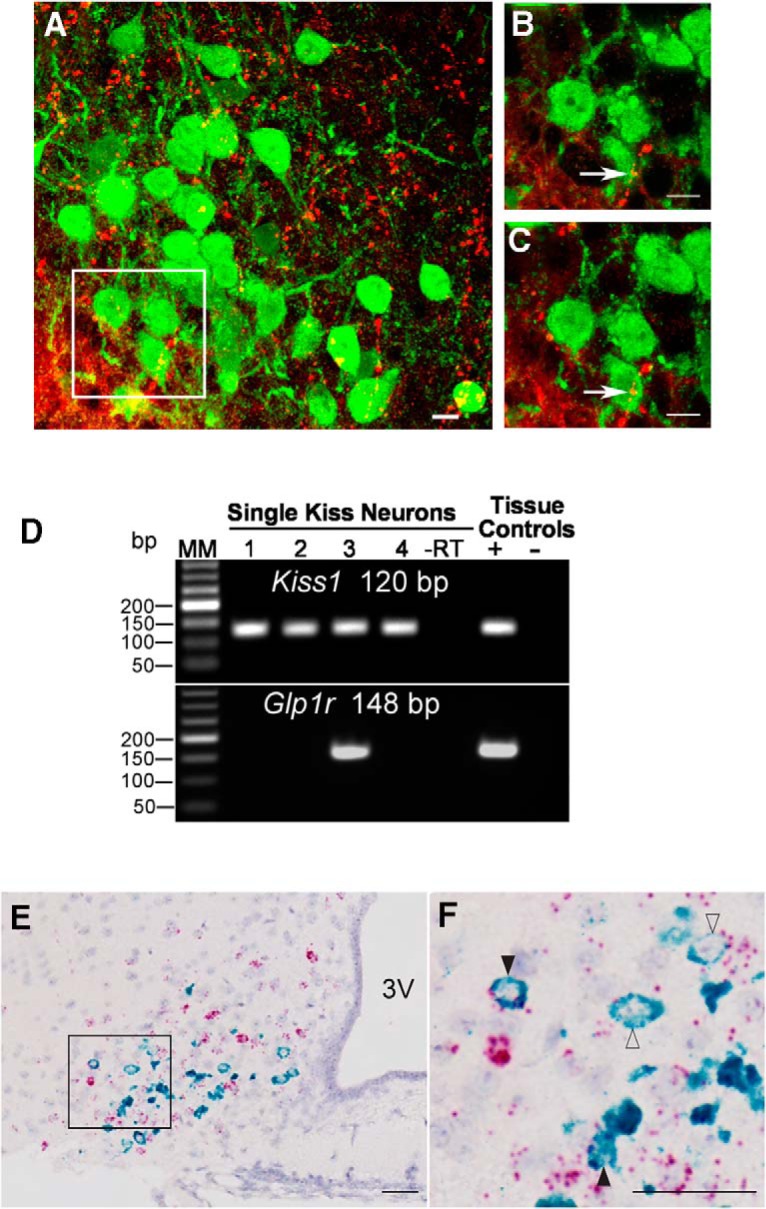
Interaction of the CNS GLP system with ARC Kiss1 in OVX mice. GLP-ir fibers (red) come in close apposition, with an average of 22% of ARC kisspeptin-ir cells (green). ***A***, Maximal projection at 40×. ***B***, Maximal projection at 63×. ***C***, 1-μm plane at 63×. Scale bars = 10 μm. *n* = 5 animals, four sections per animal. ***D***, Representative gel of single-cell RT-PCR demonstrating that a subpopulation (20%) of ARC Kiss1 cells from OVX mice express *Glp1r* mRNA. *n* = 5 animals, 16–33 cells per animal. The expected sizes for the PCR products are 120 bp for Kiss1 and 148 bp for *Glp1r*. MM, molecular marker; –RT, Kiss1-GFP cell reacted without reverse transcriptase; tissue controls (+, –), basal hypothalamic RNA reacted with (+) or without (–) RT. ***E***, Dual ISH demonstrating coexpression of *Glp1r* (red) and *Kiss1* (green) mRNA in the ARC (51 of 240 cells, 21.3% coexpression). In this example, an OVX animal showed robust *Kiss1* mRNA expression in neurons intermingled with a larger population of *Glp1r* ^+^ neurons in the ARC. ***F***, Inset of E. At higher magnification, a subpopulation of ARC Kiss1 neurons express robust and detectable mRNA signal for *Glp1r*. Filled black arrows, high *Glp1r* expression; open arrows, low *Glp1r* expression. *n* = 4 animals. Scale bars = 100 μm. 3V, third ventricle.

We used immunohistochemistry to assess GLP fiber contacts onto ARC *Kiss1-*CreGFP neurons in OXV mice (Fig. 1*A–C*). The GLP-2 primary antibody has previously been described and has complete overlap with GLP-1 distribution (Tang-Christensen et al., 2000; Vrang et al., 2007; Vrang and Grove, 2011). We determined that GLP-ir fibers come in close apposition, with an average of 22% of ARC Kiss1 cells (range 10.1%–28.6%; *n* = 5 animals, four sections per animal; Fig. 1*A–C*). We also determined that the *Glp1r* mRNA is expressed within a subpopulation (20%) of *Kiss1* cells using single-cell RT-PCR (range 18%–25%; 16–33 cells/animal; *n* = 5 animals; Fig. 1*D*). Furthermore, GLP-ir fibers come in close apposition to an average of 10.9% of GnRH cell bodies (range 8.1%–14.7%; *n* = 4 animals, five to seven sections per animal; data not shown). Our data are consistent with studies in male mice showing GLP-1-ir fiber contacts onto GnRH cells (Farkas et al., 2016).

To gain a better understanding of the neuroanatomical location of Kiss1 cells that coexpress *Glp1r* mRNA, we performed dual ISH on brain sections from OVX mice. An average of 21.3% of ARC Kiss1 neurons coexpressed *Glp1r* mRNA (Fig. 1*E*, *F*), which is consistent with our single-cell RT-PCR coexpression analysis (Fig. 1*D*). In this mixed population of *Glp1* ^+^ and *Kiss1* ^+^ neurons, we observed a higher number of *Kiss1/Glp-1r* coexpressing cells in the ventrolateral portion of the ARC (Fig. 1*F*). Taken together, these data provide neuroanatomical and molecular evidence that the CNS GLP system interacts with the reproductive neuroendocrine axis in an OVX mouse model.

### Electrophysiological recordings in ARC Kiss1 neurons treated with the GLP-1R agonist liraglutide

**Figure 2.**
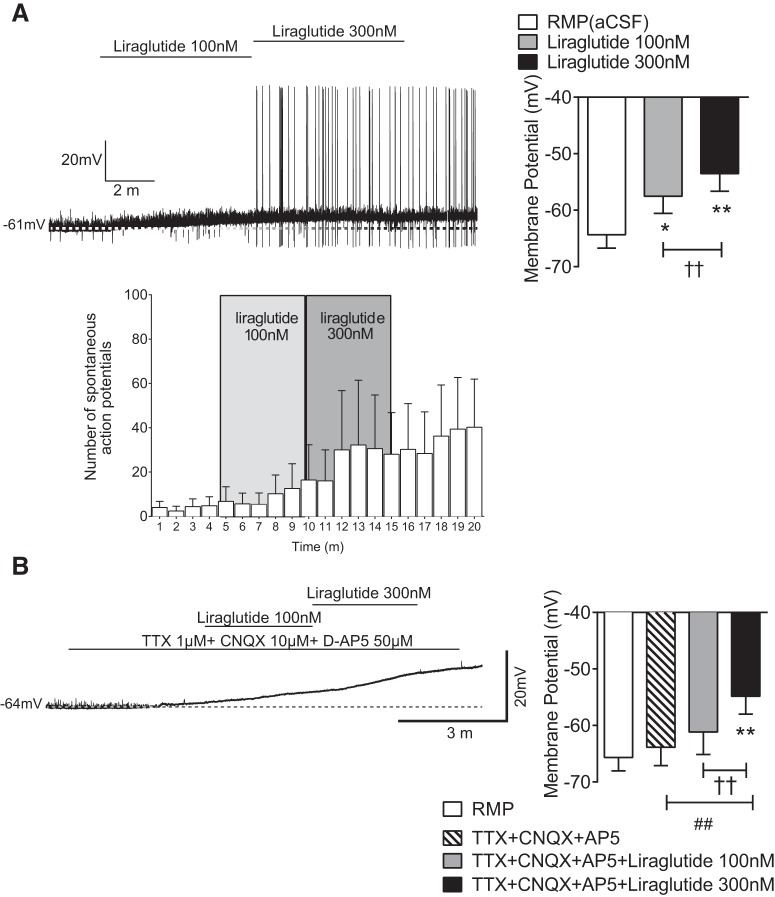
Electrophysiological recordings in brain slices demonstrating effects of GLP-1R signaling on ARC Kiss1 cells of OVX mice. ***A***, Current clamp recordings in brain slices from OVX mice demonstrate that ARC Kiss1 cells treated with liraglutide showed membrane depolarization and increased action potential firing. *p < 0.05, **p < 0.01 vs. RMP, one-way RM-ANOVA with Bonferroni’s *post hoc* test; ^††^p < 0.01, 100 vs. 300 nm liraglutide, one-way RM-ANOVA with Bonferroni’s *post hoc* test. ***B***, Current clamp recordings performed in the presence of presynaptic blockers demonstrate that liraglutide caused a membrane depolarization in ARC Kiss1 cells of OVX mice. **p < 0.01 RMP vs. TTX+CNXQ+AP5+liraglutide 300 nm, one-way RM-ANOVA with Bonferroni’s *post hoc* test; ^††^p < 0.01, 100 vs. 300 nm liraglutide, one-way RM-ANOVA with Bonferroni’s *post hoc* test. ^##^*p* < 0.01, TTX+CNXQ+AP5 vs. TTX+CNXQ+AP5+liraglutide 300 nm, one-way RM-ANOVA with Bonferroni’s *post hoc* test; *n* = 23 cells from 16 animals. ∼60% of ARC Kiss1 cells respond to liraglutide.

We determined that GLP-producing neurons come in close contact with ARC Kiss1 cells and that the GLP-1R is expressed within a subset of ARC Kiss1 neurons in OVX mice (Fig. 1). To assess the function of GLP-1R signaling within ARC Kiss1 cells, we performed current clamp recordings with application of the long-acting GLP-1R agonist, liraglutide. We found that liraglutide at 100 and 300 nm caused a membrane depolarization in ARC Kiss1 neurons (Fig. 2*A*) and an increase in action potential firing with the 300-nm concentration (Fig. 2*A*). To determine if GLP-1R signaling is directly activating ARC Kiss1 cells, we performed similar experiments in the presence of presynaptic blockers. Liraglutide at 300 nm caused membrane depolarization even in the presence of presynaptic blockers (Fig. 2*B*), which occurred in 60% of the ARC Kiss1 neurons that were tested. It should be noted that a greater percentage of ARC Kiss1 cells responded to GLP-1R agonism (∼60%) than expressed *Glp1r* mRNA (∼20%), which may reflect a greater sensitivity of electrophysiology methods compared with single-cell RT-PCR/dual ISH. Alternatively, there may be a higher level of functional GLP-1R protein at the cell surface compared with *Glp1r* mRNA expression. Nevertheless, these electrophysiological data indicate that GLP-1R signaling directly activates ARC Kiss1 cell action, suggesting that GLP-1R signaling may have a stimulatory effect on downstream GnRH/LH release.

**Figure 3.**
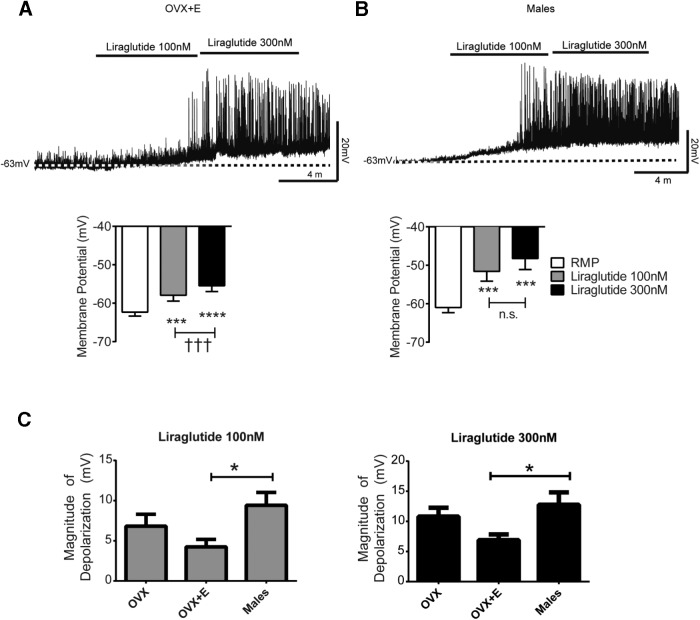
Electrophysiological recordings in brain slices demonstrating effects of GLP-1R signaling on ARC Kiss1 cells of OVX+E_2_ and male mice. ***A***, ***B***, Current clamp recordings in ARC Kiss1 cells from brain slices treated with the long-acting GLP-1R agonist, liraglutide, showed membrane depolarization in both OVX+E_2_ (***A***; 52% of cells responded) and males (***B***; 60% of cells responded). ***p < 0.001, ****p < 0.0001 vs. RMP, one-way RM-ANOVA with Bonferroni’s *post hoc* test; ^†††^*p* < 0.001, 100 vs. 300 nm liraglutide, one-way RM-ANOVA with Bonferroni’s *post hoc* test. ***C***, The magnitude of depolarization was greater in males compared with OVX+E_2_ females at 100- and 300-nm concentrations. *p < 0.05, OVX+E_2_ vs. males, one-way ANOVA with Bonferroni’s *post hoc* test. *n* = 13 male and 27 OVX +E_2_ mice.

The stimulatory effect of GLP-1R signaling on ARC Kiss1 cells in OVX mice led us to determine whether this effect is sex specific or whether estradiol modifies the action of GLP-1R signaling in ARC Kiss1 neurons. We then performed current clamp recordings in male and OVX+E_2_ *Kiss1-*CreGFP mice. ARC Kiss1 neurons from OVX+E_2_ responded to liraglutide with membrane depolarization (Fig. 3*A*); although there was a reduction in the magnitude of liraglutide-mediated responses in OVX+E_2_ compared with OVX, these differences were not significant. Male ARC Kiss1 neurons showed similar depolarization in the presence of liraglutide treatment. Overall, we observed that liraglutide caused membrane depolarization in 52% of ARC Kiss1 neurons from OVX+E_2_ mice and 60% of ARC Kiss1 neurons from males. From these data, we also determined that the initiation of spontaneous action potentials depends on the magnitude of liraglutide-mediated depolarization (Fig. 3*C*). We observed that the magnitude of liraglutide-mediated depolarization was greater in males. However, this was only statistically significant between OVX+E_2_ and male mice. As a whole, these data suggest that GLP-1R signaling activates ARC Kiss1 neurons, and this activation is not sex specific or modified by the presence of estradiol.

### Brainstem preproglucagon (Gcg) expression during calorie restriction and effect on LH levels in response to GLP-1R agonism during 48-h fasting

**Figure 4.**
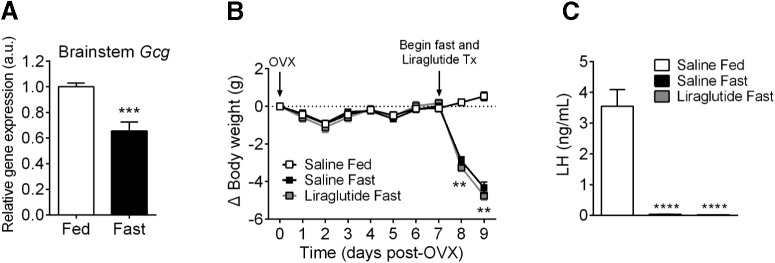
Effects of fasting in OVX mice on brainstem preproglucagon expression and on GLP-1R agonism to restore fasting-suppressed LH levels. ***A***, Brainstem preproglucagon (Gcg) expression was assessed using qPCR and was decreased after a 48-h fast (***p < 0.001, unpaired *t*-test). ***B***, ***C***, To determine whether GLP-1R agonism prevents LH inhibition during calorie restriction, liraglutide (30 nmol/kg) was administered subcutaneously twice a day at the start of a 48-h fast. Saline-fasted and liraglutide-fasted animals display decreased body weight compared with saline-fed controls (***B***; **p < 0.01, two-way ANOVA with Bonferroni’s *post hoc* test). Saline-fasted and liraglutide-fasted animals displayed significantly lower levels of LH compared with saline-fed controls (***C***; ****p < 0.0001, one-way ANOVA with Tukey’s *post hoc* test). *n* = 7–8 animals per group.

It is well established that hypothalamic Kiss1 is inhibited during fasting and calorie restriction (Luque et al., 2007; True et al., 2011). Data in male mice also indicate that brainstem preproglucagon expression is suppressed in response to prolonged fasting (Huo et al., 2008). To determine whether brainstem preproglucagon is decreased in response to fasting in OVX mice, we exposed OVX mice to a 48-h fast. Consistent with what has been observed in males, female OVX mice also have a decrease in brainstem preproglucagon expression after a 48-h fast (Fig. 4*A*; *p* = 0.0006, unpaired *t*-test). Because we determined that GLP-1R signaling stimulates ARC Kiss1 cell action in brain slices from OVX mice, we hypothesized that lack of this stimulatory signal coming from brainstem preproglucagon neurons is contributing to the downstream suppression GnRH/LH. To determine whether restoring GLP-1R signaling will relieve the inhibition of LH during fasting, we treated OVX mice with liraglutide during a 48-h fast. Liraglutide is a long-acting GLP-1R agonist that has been demonstrated to enter into the ARC upon peripheral administration (Secher et al., 2014). We took advantage of this property of liraglutide to penetrate into ARC tissue and gave twice-a-day subcutaneous injections of liraglutide during a 48-h fast in OVX mice. After the 48-h fast, body weight was significantly reduced in fasted mice treated with either saline or liraglutide compared with saline-treated fed controls (Fig. 4*B*; *p* = 0.0007, two-way ANOVA with Bonferroni’s *post hoc* test). As expected, fasted animals treated with saline experienced an inhibition of LH (Fig. 4*C*; *p* < 0.0001, one-way ANOVA with Tukey’s *post hoc* test). In contrast to what we had predicted, animals that were fasted and treated with liraglutide also experienced a similar inhibition of LH (Fig. 4*C*; *p* < 0.0001, one-way ANOVA with Tukey’s *post hoc* test), indicating that enhancing GLP-1R signaling with peripheral injections of liraglutide is not sufficient to prevent LH inhibition during fasting in OVX mice.

### Chronic ICV infusion of Ex-9 to OVX mice

**Figure 5.**
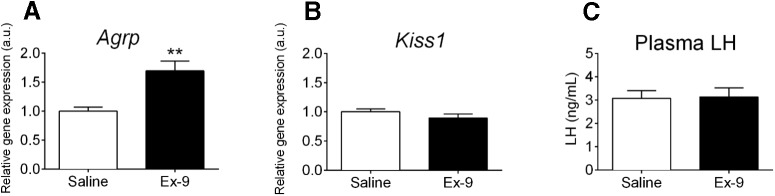
Effect of chronic ICV Ex-9 on ARC gene expression and plasma LH in OVX mice. ***A***, ***B***, One week after OVX, C57BL/6 mice received an ICV infusion of saline or Ex-9 for 6 d (7.5 nmol/d). Ex-9 caused a significant increase in ARC expression of *Agrp* (***A***; **p < 0.01, unpaired *t*-test) but did not alter ARC expression of *Kiss1* (***B***). ***C***, Plasma LH levels were similar in saline- and Ex-9–treated mice. *n* = 9 animals per group.

The data from Figs. 2, 3 and 4 suggest that although GLP-1R signaling can stimulate ARC Kiss1 action, it may not be a potent enough signal to override the inhibition on the reproductive neuroendocrine axis during extreme cases of nutrient deprivation such as a 48-h fast in mice. It could also suggest that other stimulatory signals may be more important to maintaining ARC Kiss1 action and downstream GnRH/LH release. Therefore, we next aimed to determine whether GLP-1R signaling is critical for maintaining ARC Kiss1 expression and circulating LH levels. To do this, we gave chronic ICV infusion of the GLP-1R antagonist Ex-9 to OVX mice. We chose a dose of 7.5 nmol/d of Ex-9, as this dose has been previously used in adult male mice (Nogueiras et al., 2009). After 6 d of ICV infusion of Ex-9 in OVX mice, no differences in cumulative food intake (24.62 ± 0.57 g saline vs 24.99 ± 0.46 g Ex-9; *p* = 0.62, unpaired *t*-test, *n* = 9 animals per group) were observed between saline- and Ex-9–treated animals, which is consistent with previous reports in male mice (Nogueiras et al., 2009). We did not detect a difference in body weight in Ex-9–treated animals compared with saline-treated controls; however, we did note a sizable body weight gain in both groups at the end of the infusion period (percentage increase in body weight, 12.79% ± 1.02% saline and 13.01% ± 1.39% Ex-9). The ICV implantation was started 1 week post-OVX surgery, which is about the time that mice tend to increase their body weight in response to removal of ovarian hormones (Witte et al., 2010). Therefore, the rise in body weight in response to removal of ovarian hormones may be masking the body weight effects of Ex-9 at this dose. Despite no apparent differences in body weight between saline- and Ex-9-treated animals, ARC expression of *Agrp* was increased in Ex-9-treated mice (Fig. 5*A*; *p* = 0.0023, unpaired *t*-test), confirming proper cannula placement. We did not detect differences in ARC *Kiss1* expression (*p* = 0.26, unpaired *t*-test) or plasma LH levels (*p* = 0.91, unpaired *t*-test) in Ex-9–treated animals compared with saline controls (Fig. 5*B*, *C*), suggesting that GLP-1R signaling is not essential to maintaining ARC Kiss1 and circulating LH.

## Discussion

These data are the first to provide direct neuroanatomical, molecular, and electrophysiological evidence of the interaction of the CNS GLP-1 system with ARC Kiss1 neurons to stimulate their activity. Although our studies focus on CNS-preproglucagon interactions with ARC Kiss1, we cannot discount that GLP-1 produced by the gastrointestinal tract may also activate GLP-1Rs on ARC Kiss1 neurons. Nevertheless, our reports are consistent with others demonstrating that GLP-1R signaling stimulates the reproductive neuroendocrine axis, as GLP-1 increases GnRH/LH levels in animals under normal feeding conditions (Beak et al., 1998; Outeirino-Iglesias et al., 2015). Our data suggest that GLP-1 stimulatory action on GnRH/LH may be due in part to upstream activation of ARC Kiss1 neurons. We show that the GLP-1R agonist liraglutide causes membrane depolarization in ∼60% of ARC Kiss1 neurons from OVX mice. Furthermore, we found that liraglutide depolarizes ARC Kiss1 cells from intact male and OVX+E_2_ mice, suggesting that this effect is not sex or estrogen dependent. Follow-up studies will be necessary to further characterize the pharmacological properties of GLP-1R signaling in both female and male ARC Kiss1 neurons.

In addition to acting indirectly through ARC Kiss1 neurons to modulate LH release, GLP-producing fibers come in close contact with GnRH neurons, which is consistent with the findings of other groups (Farkas et al., 2016). Furthermore, recent electrophysiological studies demonstrated that the GLP-1R agonist, exendin-4, activates GnRH neurons (Farkas et al., 2016). Although our studies focused on GLP-1R activation of ARC Kiss1 neurons, it appears that GLP-1R signaling may modify GnRH/LH release through activation of both ARC Kiss1 and GnRH neurons.

Our electrophysiological data, as well as data in the literature (Beak et al., 1998; Outeirino-Iglesias et al., 2015), describe an interaction of GLP-1R signaling with CNS Kiss1 action and downstream GnRH/LH in animals under normal energy balance, but no reports have investigated this interaction in animals under negative energy balance. Decreased circulating leptin and insulin during negative energy balance were believed to be key metabolic signals that reduced the activation of CNS kisspeptin neurons, resulting in suppressed downstream GnRH/LH release. However, previous work from our group indicates that restoration of leptin and/or insulin infused at physiological levels was not sufficient to prevent this inhibition (Xu et al., 2009; True et al., 2011). Therefore, the factors that contribute to the inhibition of the reproductive axis during negative energy balance remain elusive. Our current data reveal that GLP-1R activation stimulates ARC Kiss1 neuronal activity, leading us to hypothesize that a reduction in CNS preproglucagon may be one of these key metabolic factors. Although we do find that brainstem preproglucagon expression is reduced after a 48-h fast in OVX mice, restoring GLP-1R signaling with peripheral injections of liraglutide was not sufficient to prevent LH inhibition. Assessing GLP-1R action in electrophysiological recordings of ARC Kiss1 neurons from 48-h-fasted mice would clarify whether GLP-1R signaling has full potency during fasting. Previous data demonstrate that the anorectic action of central GLP-1R signaling is blunted in fasted rats (Sandoval et al., 2012). Furthermore, activation of GLP-1–producing neurons by cholecystokinin as measured by cfos is reduced in food deprived rats (Maniscalco and Rinaman, 2013). The decreased function of CNS GLP-1R signaling or brainstem preproglucagon activity during negative energy balance could be due to a lack of other metabolic signals necessary for full potency of action. For example, leptin is significantly reduced during nutrient deprivation (Ahrén et al., 1997), and leptin relieves the blunted anorexigenic action of CNS GLP-1R in fasted rats (Sandoval et al., 2012). Moreover, leptin prevents the suppression of brainstem preproglucagon expression in fasted mice (Huo et al., 2008), which may be due to direct action on brainstem preproglucagon neurons (Hisadome et al., 2010). In the future, studies that aim to restore multiple metabolic factors (e.g., leptin, GLP-1, insulin) may more effectively restore GnRH/LH release during negative energy balance. This multiagonist approach is currently being explored as a potential therapeutic for obesity, a disease that encompasses the dysfunction of multiple metabolic pathways (Finan et al., 2015a). Promising preclinical studies demonstrate that treating obese animal models with dual agonists (Finan et al., 2013) and triagonists (Finan et al., 2015b) could have more potent effects on weight loss than single-molecule therapies. Further investigation is necessary to determine whether a similar multiagonist therapeutic approach will ameliorate reproductive dysfunction associated with negative energy balance.

It is possible that the lack of effectiveness of GLP-1R agonism on LH levels during fasting reflects the presence of multiple inhibitory pathways that block GnRH/LH release. For example, ghrelin (Tschöp et al., 2000), corticosterone (Dallman et al., 1999), and FGF21 (Zhang et al., 2015) are all significantly elevated during food deprivation. Interestingly, all of these hormones inhibit the reproductive neuroendocrine axis (Barreiro and Tena-Sempere, 2004; Kinsey-Jones et al., 2009; Owen et al., 2013). Similarly, upregulation of brainstem glucose-sensing neurons during fasting may be overriding excitatory signals on the reproductive neuroendocrine axis. Noradrenergic glucose-sensing neurons in the A1 region of the ventral lateral medulla (VLM) are potent regulators of LH (Ritter et al., 2006), and ablation of these neurons prevents LH inhibition in response to glucoprivation (I’Anson et al., 2003). Recent studies reveal that preproglucagon-expressing neurons make close appositional contacts onto catecholaminergic neurons of the A1/C1 region of the VLM (Llewellyn-Smith et al., 2013). The physiological significance of these contacts has not been studied. It is interesting to hypothesize that under normal feeding conditions, preproglucagon-expressing neurons inhibit A1 glucose-sensing neurons. Therefore, decreased brainstem preproglucagon expression during fasting allows for the disinhibition of A1 glucose-sensing neurons in the VLM, contributing to the shutdown of the reproductive neuroendocrine axis. This interpretation is in accordance with our studies in which liraglutide did not prevent LH inhibition, as preproglucagon neurons lack GLP-1R expression and are not activated by exogenous GLP-1R agonism (Hisadome et al., 2010). Determining whether preproglucagon-expressing neurons aid in the regulation of glucose-sensing neurons in the brainstem to control proper neuroendocrine output according to metabolic status would be of interest to explore in the future.

Our present data demonstrate that pharmacological inhibition with the GLP-1R antagonist, Ex-9, does not alter ARC Kiss1 gene expression or circulating LH levels. This is consistent with transgenic mouse data demonstrating that global deletion of GLP-1R does not alter the number or distribution of gonadotrophs, and adult *Glp1r* ^–/–^ mice are fertile (MacLusky et al., 2000). Together, these data suggest that GLP-1R signaling may not be essential for maintaining ARC Kiss1 and LH in animals that are in normal energy balance. In contrast, a recent publication examined CNS GLP-1R action in prepubertal female rats and demonstrated that low doses of ICV GLP-1 synchronized vaginal opening and increased LH, whereas the GLP-1R agonist exendin-4 inhibited vaginal opening and decreased LH independently of reduced feeding (Outeirino-Iglesias et al., 2015). These data are inconsistent with our current data where pharmacological doses of a GLP-1R agonist failed to alter LH release in adult animals during fasting. The reason for this discrepancy is unclear.

Our *in vivo* pharmacological studies were all performed in OVX mice, so as to be able to measure the inhibitory effect of fasting on basal LH levels. Intact and OVX+E_2_ mice have very low levels of basal LH, making it technically difficult to measure the inhibition of LH in these models. Therefore, if GLP-1 is playing a role in the inhibition of LH due to negative energy balance, its effects should be manifested in the OVX model. Although our results show that estradiol appears to have little effect on the ability of Kiss1 cells to be activated by GLP-1R agonism, it is possible that there may be estradiol-dependent effects of GLP-1 during other reproductive states such as puberty, and follow-up studies in OVX+E_2_ models may be warranted.

In addition to regulating reproductive neuroendocrine function, Kiss1r signaling may regulate energy homeostasis, as loss of Kiss1r signaling leads to body weight gain (Tolson et al., 2014). The kisspeptin population responsible for this effect is unknown, but ARC Kiss1 neurons may be prime candidates, as they are in a primary brain area that regulates energy homeostasis and send fiber projections to numerous hypothalamic nuclei that regulate feeding and energy expenditure (Yeo and Herbison, 2011; Yeo, 2013). Furthermore, kisspeptin-ir neurons are in close apposition with ARC proopiomelanocortin (POMC) neurons, and electrophysiological recordings demonstrate that kisspeptin directly excites ARC POMC and indirectly inhibits ARC NPY neurons (Fu and van den Pol, 2010). Although GLP-1R signaling was reported to regulate GnRH/LH release (Beak et al., 1998; Outeirino-Iglesias et al., 2015), the most consistent physiological output of GLP-1 mimetics is reduced body weight, which requires CNS GLP-1R signaling (Sisley et al., 2014). Activation of GLP-1R signaling directly stimulates ARC POMC neurons and indirectly inhibits ARC NPY neurons, which are thought to be important mechanisms whereby GLP-1R agonists mediate a reduction in body weight (Secher et al., 2014). Our electrophysiological data may suggest that, in addition to regulating ARC POMC and NPY, GLP-1R signaling regulates energy homeostasis through activation of ARC Kiss1 neurons. Determining the effectiveness of GLP-1R agonists on weight loss in transgenic animals with a specific inhibition of ARC Kiss1 neurons may help to clarify this role of ARC Kiss1.

In summary, we find that GLP-producing fibers interact with ARC Kiss1 cells that express GLP-1R. Furthermore, GLP-1R signaling directly activates ARC Kiss1 function in an estradiol-independent manner. Despite a clear stimulatory effect on ARC Kiss1 action, we find that pharmacological activation of GLP-1R signaling during fasting or pharmacological inhibition of CNS GLP-1R signaling during normal feeding does not alter circulating LH levels, suggesting that GLP-1R activation is not critical for the maintenance of LH in adult animals. Alternatively, GLP-1R signaling within ARC Kiss1 cells may regulate an unidentified physiological output of ARC Kiss1 activation. Further studies are necessary to fully understand the significance of GLP-1R activation of ARC Kiss1. Collectively, these data not only identify a novel signal that stimulates ARC Kiss1 cell activity, but also highlight the complexity of metabolic signals that regulate the reproductive neuroendocrine axis.

## Synthesis

Reviewing Editor: Jeffrey Blaustein, University of Massachusetts Decisions are customarily a result of the Reviewing Editor and the peer reviewers coming together and discussing their recommendations until a consensus is reached. When revisions are invited, a fact-based synthesis statement explaining their decision and outlining what is needed to prepare a revision will be listed below.

accepted as is
